# The impact of specialized pediatric palliative care on advance care planning and healthcare utilization in children and young adults: a retrospective analysis of medical records of in-hospital deaths

**DOI:** 10.1186/s12904-024-01448-w

**Published:** 2024-05-22

**Authors:** Cho Hee Kim, Jung Lee, Ji Weon Lee, Min Sun Kim

**Affiliations:** 1 College of Nursing, Kangwon National University, Chuncheon, Republic of Korea; 2 Integrative Care Hub, Seoul National University Hospital, 101 Daehak-ro, Jongno-gu, Seoul, 03080 Republic of Korea; 3 Department of Pediatrics, Seoul National University Hospital, Seoul, Republic of Korea

**Keywords:** Palliative care, Advance care planning, Delivery of health care, Health care cost, Retrospective studies

## Abstract

**Background:**

Pediatric palliative care supports children and young adults with life-limiting conditions and their families, seeking to minimize suffering and enhance quality of life. This study evaluates the impact of specialized palliative care (SPC) on advance care planning (ACP) and patterns of end-of-life care for patients who died in the hospital.

**Methods:**

This is a retrospective cohort study of medical records extracted from a clinical data warehouse, covering patients who died aged 0–24 in an academic tertiary children’s hospital in South Korea. Participants were categorized into before (2011–2013; pre-period) and after (2017–2019; post-period) the introduction of an SPC service. Within the post-period, patients were further categorized into SPC recipients and non-recipients.

**Results:**

We identified 274 and 205 patients in the pre-period and post-period, respectively. ACP was conducted more and earlier in the post-period than in the pre-period, and in patients who received palliative care than in those who did not. Patients who received SPC were likely to receive less mechanical ventilation or cardiopulmonary resuscitation and more opioids. A multivariable regression model showed that earlier ACP was associated with not being an infant, receiving SPC, and having a neurological or neuromuscular disease.

**Conclusions:**

SPC involvement was associated with more and earlier ACP and less intense end-of-life care for children and young adults who died in the hospital. Integrating palliative care into routine care can improve the quality of end-of-life care by reflecting patients’ and their families’ values and preferences.

**Supplementary Information:**

The online version contains supplementary material available at 10.1186/s12904-024-01448-w.

## Background

Pediatric palliative care is an additional layer of support for infants, children, adolescents, and young adults living with life-limiting conditions, along with their families, aiming to minimize physical, psychosocial, and spiritual suffering and enhance quality of life [1]. As an integrative model of pediatric palliative care, specialized palliative care (SPC) is provided by a multidisciplinary team of professionals to children and families with more complex care needs [2]. Advance care planning (ACP), a key element of palliative care, involves anticipatory discussion and healthcare decision-making. ACP is not limited to life-sustaining treatment decisions at the end-of-life; it is a systematic, family-centered conversation to reflect the patient’s and family’s values and preferences into goals of care [3].

Palliative care and ACP discussion often result in patients using less inpatient and intensive care and more outpatient, community, and home-based services during the end-of-life in the adult population, which has been the focus of previous research [4, 5]. Palliative care is not limited to end-of-life care or intended to control healthcare use and costs. However, not assessing the patients’ and families’ preferences for care may lead to increased hospitalization at the end-of-life, undermining the goal concordant care, one of the important quality indicators of successful ACP [6]. ACP is also crucial for children and young adults, as those with life-limiting conditions require multiple specialized healthcare and complicated symptom management, even at the end-of-life [7].

Several studies on the benefits of pediatric SPC have been published in recent years, reporting mixed results on the impact on the use of acute healthcare and intensive medical treatments at the end-of-life [2]. Regarding impact on ACP, patients with SPC showed a higher percentage of ACP discussion [8], and they initiated ACP earlier [9, 10]. As for healthcare use, previous studies reported that, in children who died of cancer, SPC was associated with reduced intensive care unit (ICU) admissions and less intensive end-of-life care [11], fewer invasive procedures, and fewer deaths in the ICU [12]. Other studies examining children’s deaths in a children’s hospital suggested that the acute hospital utilization is more affected by the proximity to death and expected deterioration than by the SPC involvement [13]. In addition, few studies included a wide range of ages or disease groups, making it difficult to understand the overall impact of palliative care for children and adolescents.

Therefore, this study aimed to explore the impact of SPC on children and young adults who died while hospitalized in a single institution. Specifically, we examined the impact of SPC on the patterns of decision-making, end-of-life care, and healthcare use during the patient’s last month of life by comparing before and after the implementation of palliative care. Moreover, we compared patients with and without palliative care.

## Methods

### Study design

A retrospective review of medical records was conducted at Seoul National University Children’s Hospital (SNUCH). The ethics committee of the Seoul National University Hospital (registration number 2010-112-1165) waived the requirement to obtain written informed consent, as this was a retrospective study. This study was reported in accordance with the REporting of studies Conducted using Observational Routinely collected health Data (RECORD) recommendations [14].

### Setting

SNUCH, the largest pediatric tertiary care center in South Korea (317 beds; approximately 1,000 daily outpatient visits), launched a Dreamseeds Center, an SPC service, in 2014. The center provides consultation services and outpatient clinics by a multidisciplinary team of physicians (a palliative care physician and a psychiatrist), nurses, a social worker, expressive therapists (art, movement, play), and more. SPC begins when a patient’s primary physician makes a referral. The center provides care services for inpatients and outpatients, including pain and symptom management, communication and decision-making support, care coordination, emotional and social support, art therapy, and bereavement care. In addition, telephone counseling and need-based home visits are provided for patients at home during end-of-life.

### Participants

This study included all patients aged < 25 years who were treated and died at SNUCH in two different periods, before or after the implementation of palliative care: (a) pre-period (1 January 2011 to 31 December 2013) and (b) post-period (1 January 2017 to 31 December 2019). These periods were selected to account for the time it takes for acculturation of integrated palliative care at the institutional level.

### Sources

Patients were identified as having the status of “death” at discharge, and then data were retrieved from SNUCH’s clinical data warehouse (SUPREME; Seoul National University Hospital Patient Research Environment, https://supreme.snuh.org). Information on healthcare use and costs for inpatients, outpatients, and emergency services was obtained from institutional administrative data. Finally, the SNUCH palliative care registry was queried to see if the patient received palliative care. The SNUCH palliative care registry is an independent database containing detailed information on care plans and service provisions for each patient and family. Data were linked using hospital patient identifiers and dates of birth as individual-level indicators. Then, SPC professionals (a pediatric palliative physician, a psychiatrist, two nurses, and a social workers) reviewed the patient’s health records and/or SNUCH palliative registry to identify the ACP variables. Data were collected and linked between October and December 2021.

### Variables

We collected patients’ demographic and clinical characteristics (date of birth, sex, insurance type, residential address, primary diagnoses, treatment duration, date of death, and location of death) and SPC enrollment. Treatment duration was defined as months from diagnosis to death based on the primary diagnosis of the last hospitalization. We defined ACP at three levels; if a preference or plan for future care was recorded in the medical records, we categorized it as “discussed” and collected the date to generate “the days from ACP initiation to death”; if wishes for life-sustaining treatment (LST) (cardiopulmonary resuscitation [CPR], hemodialysis, mechanical ventilation, chemotherapy which are specified by the law) are recorded in the medical records but there was no completed legal document, we categorized it as “medical documentation on LST;” if there was a legal document on wishes for LST, we categorized as “legal documentation on LST.” Furthermore, end-of-life care characteristics during the last month of life were collected, including the number of hospitalizations, length of stay, use of intensive treatments (mechanical ventilation, CPR, ICU admission, and ICU days), inpatient costs, number of outpatient department (OPD) visits, and ED visits. Admission following an ED visit was categorized as admission, as the data were not distinguishable; therefore, ED visits could be underestimated in this study. Variables of pediatric ICU admission rates and days were generated to analyze ICU utilization only with physical deterioration, excluding patients who were born and stayed in the neonatal intensive care unit (NICU) until death. The primary diagnoses were categorized using the complex chronic condition (CCC) classification (version 2) based on the International Classification of Disease, 10th edition [15], using the “pccc” R package [16]. Once a patient was referred to the SPC service but died outside the hospital, demographic and clinical characteristics were extracted from the SNUCH palliative care registry.

### Statistical methods

Descriptive statistics were generated, including means with standard deviations and medians with interquartile ranges for continuous variables, and frequencies and percentages for categorical variables. Demographic and clinical characteristics of patients who died before the introduction of SPC (pre-period) were compared with those who died after (post-period). To examine the impact of SPC on end-of-life care, we compared patterns of end-of-life care for patients who received palliative care (SPC group) and those who did not receive palliative care (non-SPC group) using Fisher’s exact test and the Wilcoxon rank sum test for categorical or continuous variables, respectively. Multivariable logistic regression analysis of key end-of-life care characteristics (completion of legal ACP document, opioid use, death in the general ward, mechanical ventilation, CPR, and ICU admission) was followed to investigate adjusted odds ratios with post-period or SPC as an independent variable controlling confounding factors. Finally, a multivariable linear regression model was fitted to identify the associations between demographic and clinical characteristics and days from the initial ACP to death. Log transformation of the days from the initial ACP to death was performed to normalize the residuals in the regression analysis. Variables with marginally significant associations (*p* < .10) in univariable analysis were included in the multivariable model. Data were analyzed using R version 4.0.2.

## Results

### Demographic and clinical characteristics

**Fig. 1:**
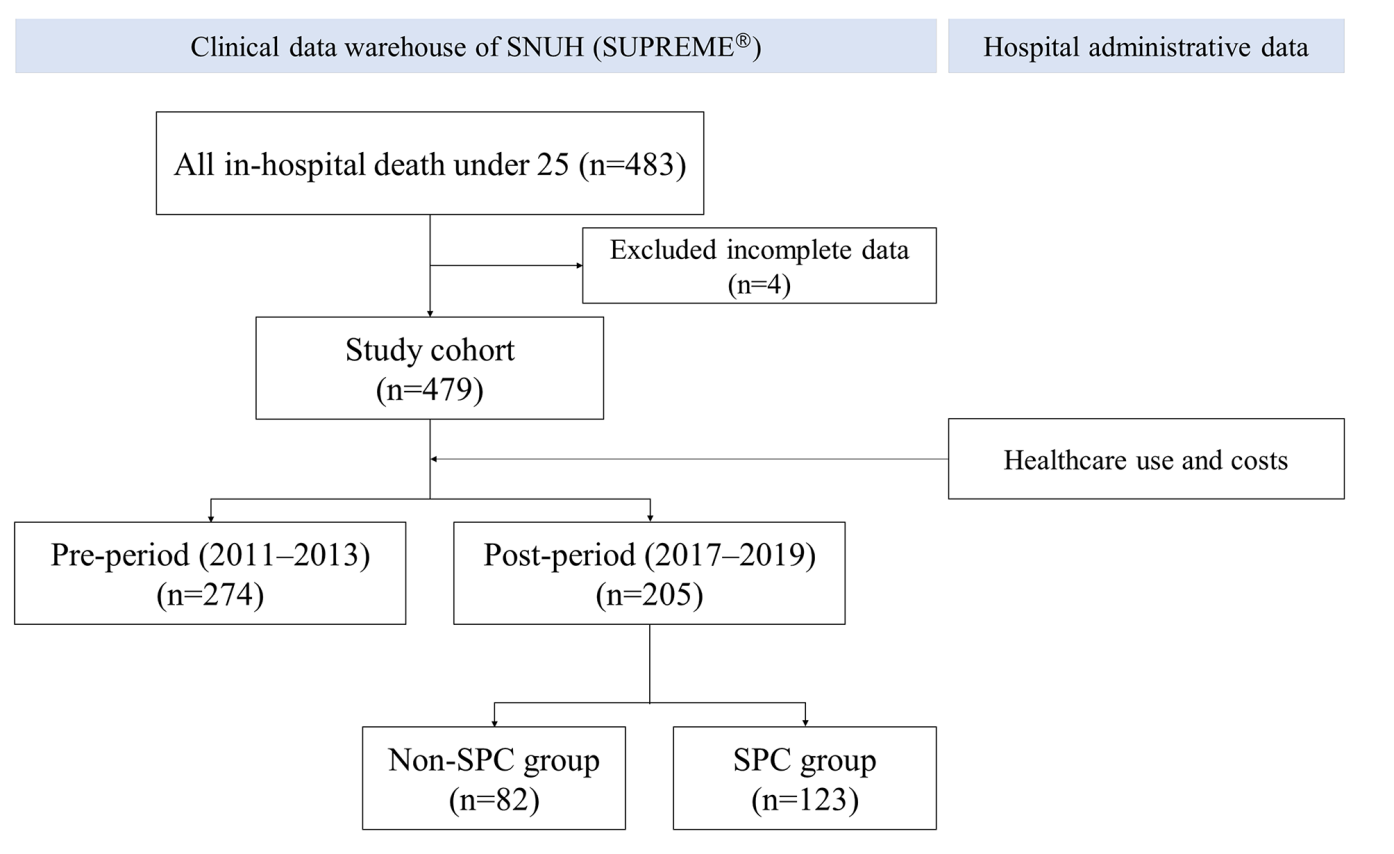
Flow chart of data sources and study cohort linkages. SPC, specialized palliative care

Our analysis identified 479 patients aged < 25 years who died in the hospital; of these, 205 (42.8%) deaths occurred during the post-period, and 123 patients were enrolled in SPC (60%) (Fig. 1). No statistically significant difference was found in the demographic and clinical characteristics between pre- and post-periods except the proportion of hematological or immunological condition (Table 1). The non-SPC and SPC groups were similar in demographic characteristics, although the SPC group had a higher mean age. A larger proportion of patients with malignancy (*p* < .001), congenital or genetic (*p* = .007), neurologic and neuromuscular (*p* = .044), hematological or immunological condition (*p* < .001) received SPC, whereas a larger proportion of patients with premature and neonatal condition did not (*p* < .001). Multiple CCCs was similar between the pre- and post-period; however, a larger proportion of patients with two or more CCC received SPC (*p* < .001). The SPC group showed longer treatment duration than the non-SPC group, which might be explained by differences in CCC categories, particularly neurological and neuromuscular conditions.

**Table 1:** Demographic and clinical characteristics of children and young adults who died in the children’s hospital (*n* = 479)

Characteristics	Total (*n* = 479)		Pre-period (2011–2013) (*n* = 274))			Post-period (2017–2019) (*n* = 205)			*p*-value	Post-period (2017–2019)						
										Non-SPC group (*n* = 82)			SPC group (*n* = 123)			*p*-value
			*N* (%), mean ± SD, or median (IQR)							*N* (%), mean ± SD, or median (IQR)						
Sex																
Female	194	(40.5)	115	(42.0)		79	(38.5)		0.449	36	(43.9)		43	(35.0)		0.197
Male	285	(59.5)	159	(58.0)		126	(61.5)			46	(56.1)		80	(65.0)		
Age at death, year	4.5	± 6.3	4.0	± 5.9	1 (0,7)	5.1	± 6.8	1 (0,10)	0.266	2.5	± 5.4	0 (0, 0)	6.8	± 7.1	4 (0, 13)	< 0.001
Insurance																
NHI	457	(95.4)	262	(95.6)		195	(95.1)		0.822	78	(95.1)		117	(95.1)		1.000
Other	22	(4.6)	12	(4.4)		10	(4.9)			4	(4.9)		6	(4.9)		
Resident area																
Metropolitan near the hospital	338	(70.6)	199	(72.6)		139	(67.8)		0.252	60	(73.2)		79	(64.2)		0.179
Other	141	(29.4)	75	(27.4)		66	(32.2)			22	(26.8)		44	(35.8)		
CCC diagnoses^a^																
Cardiovascular	182	(38.0)	110	(40.1)		72	(35.1)		1.000	28	(34.1)		44	(35.8)		1.000
Malignancy	136	(28.4)	79	(28.8)		57	(27.8)		1.000	4	(4.9)		53	(43.1)		< 0.001
Congenital or genetic	87	(18.2)	59	(21.5)		28	(13.7)		0.297	3	(3.7)		25	(20.3)		0.007
Premature or neonatal	86	(18.0)	41	(15.0)		45	(22.0)		0.535	32	(39.0)		13	(10.6)		< 0.001
Neurological and neuromuscular	74	(15.4)	34	(12.4)		40	(19.5)		0.366	8	(9.8)		32	(26.0)		0.044
Hematological or immunological	74	(15.4)	18	(6.6)		56	(27.3)		< 0.001	9	(11.0)		47	(38.2)		< 0.001
Metabolic	53	(11.1)	33	(12.0)		20	(9.8)		1.000	8	(9.8)		12	(9.8)		1.000
Gastrointestinal	51	(10.6)	24	(8.8)		27	(13.2)		1.000	11	(13.4)		16	(13.0)		1.000
Renal	41	(8.6)	20	(7.3)		21	(10.2)		1.000	5	(6.1)		16	(13.0)		1.000
Respiratory	38	(7.9)	17	(6.2)		21	(10.2)		1.000	8	(9.8)		13	(10.6)		1.000
Number of CCCs									0.087							< 0.001
0	45	(9.4)	30	(10.9)		15	(7.3)			12	(14.6)		3	(2.4)		
1	195	(40.7)	121	(44.2)		74	(36.1)			40	(48.8)		34	(27.6)		
2	134	(28.0)	69	(25.2)		65	(31.7)			18	(22.0)		47	(38.2)		
> 3	105	(21.9)	54	(19.7)		51	(24.9)			12	(14.6)		39	(31.7)		
Treatment duration, month	22.3	± 48.1	20.1	± 43.6	2 (0,15)	25.1	± 53.6	4 (0,1)	0.138	16.3	± 48.6	0 (0, 2)	31.0	± 56.2	11 (2, 28)	< 0.001

SPC, specialized palliative care; SD, standard deviation; IQR, interquartile range; NHI, National Health Insurance; CCC, complex chronic condition

^a^ Multiple responses were allowed

During the post-period, 59 patients received SPC but died outside the hospital; therefore, these patients were excluded from the analysis. Patients who died outside the hospital were older than those who died in the hospital (median age of 9 years vs. 4 years, *p* = .001) and were less likely to have cardiovascular (35.8% vs. 15.3%, *p* = .007), congenital or genetic (20.3% vs. 6.8%, *p* = .034), and hematological or immunological condition (38.2% vs. 15.3%, *p* = .003) (Supplementary Table 1). The analysis did not include the location of death for patients who died outside the hospital due to incomplete data, which included other hospitals, home, and unknown places.

### Comparison of before and after the implementation of specialized palliative care

In the post-period, more patients engaged in ACP, and they were less likely to receive intensive care at the end-of-life compared to those in the pre-period (Table 2). More patients in the post-period engaged in ACP and completed medical and legal documents than those in the pre-period. Days from initial ACP to death were longer in the post-period, indicating earlier ACP discussion. During the last month of life, patients who died in the post-period were less likely to receive mechanical ventilation, cardiopulmonary resuscitation, and more likely to receive opioids. After controlling sex, being infant, residence, insurance type, CCC categories and number of CCCs, patients who died in the post-period were more likely to complete ACP legal document (adjusted odds ratio [aOR] 1.62, 95% CI 1.08 to 2.46), use opioids (aOR 1.89, 95% CI 1.19 to 3.06) and less likely to receive mechanical ventilation (aOR 0.37, 95% CI 0.19 to 0.68), and CPR (aOR 0.45, 95% CI 0.30 to 0.68) (Supplementary Table 2).

**Table 2:** Pattern of advance care planning and end-of-life care in the last month of life (*n* = 479)

Characteristics	Total (*n* = 479)		Pre-period (2011–2013) (*n* = 274)			Post-period (2017–2019) (*n* = 205)			*p*-value
	*N* (%), mean ± SD, or median (IQR)								
Palliative care consultation			0	(0.0)		123	(60.0)		< 0.001
Advance care planning									
Discussed	365	(76.2)	196	(71.5)		169	(82.4)		0.006
Days from ACP initiation to death	24.9	± 66.1	13.9	± 49.3	0 (0, 6)	37.6	± 79.3	6 (1, 33)	< 0.001
Medical documentation on LST	276	(57.6)	123	(44.9)		153	(74.6)		< 0.001
Legal documentation on LST	228	(47.6)	116	(42.3)		112	(54.6)		0.022
Treatment in the last month of life									
Mechanical ventilation	390	(81.4)	235	(85.8)		155	(75.6)		0.005
Oxygen therapy	290	(60.5)	213	(77.7)		77	(37.6)		< 0.001
HD or PD	134	(28.0)	64	(23.4)		70	(34.1)		0.009
Transfusions	362	(75.6)	204	(74.5)		158	(77.1)		0.509
Antibiotics	410	(85.6)	230	(83.9)		180	(87.8)		0.240
Opioids (IV, PO, or patch)	338	(70.6)	177	(64.6)		161	(78.5)		0.001
CPR	229	(47.8)	154	(56.2)		75	(36.6)		< 0.001
Chemotherapy (*n* = 156)^a^	42	(26.9)	21	(24.4)		21	(30.0)		0.434
Location of death									0.588
Pediatric ICU	198	(41.3)	117	(42.7)		81	(39.5)		
Neonatal ICU	153	(31.9)	90	(32.8)		63	(30.7)		
General ward	107	(22.3)	55	(20.1)		52	(25.4)		
ED	21	(4.4)	12	(4.4)		9	(4.4)		

SD, standard deviation; IQR, interquartile range; LST, life-sustaining treatment; HD, hemodialysis; PD, peritoneal dialysis; IV, intravenous; PO, per oral; CPR, cardiopulmonary resuscitation; ICU, intensive care unit; ED, emergency department

^a^ The proportion of patients who had ever received chemotherapy was calculated using the number of patients with malignancy or hematologic and immunologic conditions as a denominator

### Comparison between patients who received specialized palliative care and those who did not

Patients in SPC group were more likely to engage in ACP and were less likely to receive intensive care at the end-of-life than those in non-SPC group (Table 3). Patients enrolled in SPC had a higher proportion of ACP and legal documentation. ACP occurred earlier in the SPC group than in the non-SPC group. Additionally, patients in the SPC group were less likely to be mechanically ventilated, more likely to receive opioids, and less likely to receive CPR. The proportions of patients that received transfusion, antibiotics, or chemotherapy were similar, regardless of SPC involvement. In both the SPC and non-SPC groups, over half of the patients died in the ICU (pediatric intensive care unit [PICU] or NICU) with PICU days was longer in SPC group. However, 49 patients enrolled in SPC (39.8%) died in the general ward, compared with only three patients (3.7%) who were not enrolled. The multivariable regression analysis revealed that the SPC group remained a significant factor in explaining the completion of ACP legal documentation (aOR 5.47, 95% CI 2.53 to 12.31), opioids (aOR 19.18, 95% CI 5.64 to 82.61), and CPR (aOR 0.18, 95% CI 0.08 to 0.41) after controlling for confounding factors, including sex, being an infant, CCC categories, and number of CCCs (Supplementary Table 3).

**Table 3:** Pattern of advance care planning, end-of-life care, and healthcare utilization in the last month of life in the post-period (*n* = 205)

Characteristics	Total (*n* = 205)		Non-SPC group (*n* = 82)			SPC group (*n* = 123)			*p*-value
	*N* (%), mean ± SD, or Median (IQR)								
Advance care planning									
Discussed	169	(82.4)	53	(64.6)		116	(94.3)		< 0.001
Days from ACP initiation to death	37.6	± 79.3	4.8	± 14.3	0 (0, 2)	52.6	± 91.5	16 (3, 63)	< 0.001
Medical documentation on LST	153	(74.6)	58	(70.7)		96	(78.0)		0.294
Legal documentation on LST	112	(54.6)	24	(29.3)		88	(71.5)		< 0.001
Treatment at the last month of life									
Mechanical ventilation	155	(75.6)	76	(92.7)		79	(64.2)		< 0.001
Oxygen therapy	77	(37.6)	15	(18.3)		62	(50.4)		< 0.001
HD or PD	70	(34.1)	29	(35.4)		41	(33.3)		0.029
Transfusions	158	(77.1)	61	(74.4)		97	(78.9)		0.456
Antibiotics	180	(87.8)	70	(85.4)		110	(89.4)		0.384
Opioids (IV, PO, or patch)	161	(78.5)	49	(59.8)		112	(91.1)		< 0.001
CPR	75	(36.6)	49	(59.8)		26	(21.1)		< 0.001
Chemotherapy (*n* = 70)^a^	21	(30.0)	3	(42.9)		18	(28.6)		0.421
Location of death									< 0.001
Pediatric ICU	81	(39.5)	30	(36.6)		51	(41.5)		
Neonatal ICU	63	(30.7)	44	(53.7)		19	(15.4)		
General ward	52	(25.4)	3	(3.7)		49	(39.8)		
ED	9	(4.4)	5	(6.1)		4	(3.3)		
Hospital admissions									
Hospital days	18.5	± 12.0	12.6	± 12.3	6, (1.5, 30)	22.2	± 10.2	30 (15, 30)	< 0.001
ICU admissions	149	(72.7)	73	(89.0)		76	(61.8)		< 0.001
ICU days	14.8	± 12.1	10.7	± 11.4	4 (2, 19)	18.8	± 11.5	23.5 (7, 30)	< 0.001
Pediatric ICU admissions, yes	87	(42.4)	30	(36.6)		51	(41.5)		0.484
Pediatric ICU days	17.3	± 12.2	13.8	± 12.5	7 (2, 30)	19.2	± 11.7	26 (7, 30)	0.040
Cost for inpatient service (USD)									
Inpatient cost, total	30370.7	± 27066.2	28010.3	± 30842.8	12588.9 (5787.5,41700.8)	31865.7	± 24469.7	25020.5 (10936.6, 48152.6)	0.043
Copayment	3911.1	± 5455.5	3074.0	± 3773.8	1579.9 (442.4, 4362.8)	4436.9	± 6244.1	3084.9 (1305.0, 5584.8)	0.003
Cost, insured	3416.4	± 5508.2	3158.2	± 4352.8	2037.5 (599.1, 4123.7)	3578.5	± 6135.5	1885.0 (745.5, 4275.1)	0.599
Cost per day	2159.2	± 1833.3	3123.0	± 2221.9	2646.7 (1578.3, 3907.2)	1553.8	± 1202.5	1299.4 (685.8, 1992.9)	< 0.001
OPD visits									
Visited OPD at least once	54	(26.3)	13	(15.9)		41	(33.3)		0.005
no. of OPD visits (*n* = 54)	2.1	± 1.9	2.2	± 2.6	1 (1, 2)	2.1	± 1.6	1 (1, 2)	0.517
ED visits^b^									
Visited ED at least once	20	(9.8)	8	(9.8)		12	(9.8)		1.000
No. of ED visits (*n* = 20)	1.2	± 0.4	1.0	± 0.0	1 (1, 1)	1.3	± 0.5	1 (1, 1.5)	0.386

SPC, specialized palliative care; SD, standard deviation; IQR, interquartile range; LST, life-sustaining treatment; HD, hemodialysis; PD, peritoneal dialysis; IV, intravenous; PO, per oral; CPR, cardiopulmonary resuscitation; ICU, intensive care unit; ED, emergency department; OPD, outpatient department

^a^ The proportion of patients who had ever received chemotherapy was calculated using the number of patients with malignancy or hematologic and immunologic condition as a denominator

^b^ Cases of ED visits resulted in admission excluded and were categorized as usage of admission

Regarding healthcare use in the last month of life, patients enrolled in SPC showed more hospital and ICU days. While the PICU admission rates were comparable between the SPC and non-SPC groups, the former had more PICU days. In addition, the SPC group accrued higher total costs for inpatient services; however, the cost per inpatient each day was lower in the SPC group. Patients enrolled in SPC were more likely to visit the OPD during the last month of life, relative to non-SPC patients; however, the two groups had similar numbers of visits to the OPD or ED.

### Factors associated with early advance care planning prior to death

**Fig. 2:**
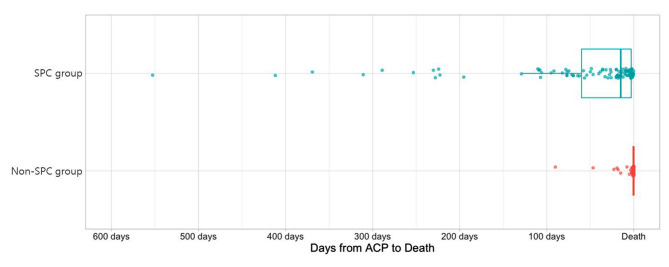
Days from ACP initiation to death in post-period (2017–2019) according to SPC involvement. Dot plots override box-plot diagrams. SPC, specialized palliative care; ACP, advance care planning

The days from the initial ACP to death was analyzed to identify factors associated with early engagement in ACP. Among 169 patients who discussed ACP, average days from initial ACP to death was 37.6 days (standard deviation 79.3 days). Supplementary Table 4 depicts the descriptive statistics of days from the initial ACP to death. A multivariable linear regression model controlling for sex, being an infant, residential area, insurance type, malignancy, and neurological and neuromuscular condition was conducted (Table 4). SPC involvement was associated with more days from initial ACP to death, indicating earlier ACP (β 1.44, 95% CI 0.89 to 1.99, *p* < .001) (Fig. 2), while being an infant was negatively associated with earlier ACP (β -0.74, 95% CI -1.28 to -0.19, *p* = .008). Being diagnosed with a neurological and neuromuscular condition was associated with more days from the initial ACP to death (β 0.76, 95% CI 0.17 to 1.35, *p* = .012).

**Table 4:** Factors associated with days from initial discussion of advance care planning to death (*n* = 169)

Characteristics	β	(95% CI)		*p*-value
Intercept	1.13	(0.45	to 1.82)	< 0.001
Male^a^	-0.11	(-0.57	to 0.34)	0.624
Infant^a^	-0.74	(-1.28	to -0.19)	0.008
Residence				
Others	reference			
Metropolitan near the hospital	0.23	(-0.24	to 0.69)	0.335
Insurance type				
Others	reference			
NHI	0.35	(-0.76	to 1.45)	0.538
SPC involvement				
Non-SPC group	reference			
SPC-group	1.44	(0.89	to 1.99)	< 0.001
Malignancy^a^	0.42	(-0.19	to 1.02)	0.174
Neurological and Neuromuscular^a^	0.76	(0.17	to 1.35)	0.012

Adjusted R^ = 0.343, F = 11.98, *p* = < 0.001

CI, confidence interval; NHI, national health insurance; SPC, specialized palliative care

^a^ reference group = no

## Discussion

### Main findings

To our knowledge, this is one of the first studies to examine the impact of SPC on children and young adults who died in a tertiary children’s hospital, with particular attention on ACP. This retrospective analysis compared the periods before and after palliative care implementation and those who received SPC and those who did not. The results demonstrated that patients who received SPC were more likely to have ACP and initiate discussions earlier. Furthermore, patients who received SPC were less likely to receive highly intensive care during the last month of life, including mechanical ventilation, CPR, and dialysis, and more likely to receive opioids.

### Initiation of advance care planning

Facilitating ACP is a crucial role of palliative care, providing treatment and care in line with the values and preferences of patients and their families. Early initiation of discussions about the goals of care and routine revisiting of the care plan may improve patients’ and families’ experiences without increasing distress, strain, or emotional burden [17]. We found that patients who received SPC were more likely to engage in ACP, complete medical and legal documents on life-sustaining treatments, and even engage earlier than those who did not. This is meaningful considering the policy in South Korea that limits legal documentation to patients expected to have imminent death, despite the difficulty in clearly defining “imminent death” [18]. This trend to more frequent and earlier ACP was also observed in the post-period than in the pre-period. Our findings are consistent with a previous retrospective cohort study in the U.S., which investigated changes in ACP among children with cancer at the end of life through historical comparisons [19]. Additionally, our study demonstrated an improvement in ACP during the post-period following the implementation of SPC, even in pediatric patients with conditions other than cancer. A possible explanation for this could be that the implementation of SPC within a healthcare institution not only improves the quality of care for patients and families enrolled in SPC but also enhances the culture within the entire institution, allowing a general palliative approach. Our finding of increased opportunities for early ACP among patients with SPC highlights the role of palliative care in setting the goals of care for patients and their families.

Our results showed that ACP would likely be delayed if the patient was an infant. Previous qualitative research of NICU healthcare professionals reported that ACP was challenging owing to the uncertain prognosis of infants and various possible options for advanced medical treatment [20]. Nonetheless, parents found that routine ACP, rather than a startling or desperate event, and standardized psychosocial support helped make end-of-life decisions for high-risk infants [21]. Several factors could facilitate systematic early ACP, including designated personnel, professional awareness, and knowledge of ACP [22]. Further research is needed to address how to standardize ACP for neonates and infants.

### Use of intensive care at the end-of-life

Our results showed that patients with SPC received less intensive care such as less mechanical ventilation and CPR, and more opioids during the last month of life. These results were consistent with previous studies indicating higher inpatient service use among children with cancer, yet among them, patients with SPC received less intensive end-of-life care [10, 23]. However, patients with SPC had longer PICU stays and more PICU deaths in the present study, which is inconsistent with previous studies [10]. In conjunction with our findings, recent studies have also presented mixed results regarding the impact of palliative care on end-of-life ICU utilization [24, 25]. Subsequent investigations are required to examine whether these observations indicate goal-concordant care or are influenced by systemic factors, such as the timing of palliative care referrals [25]. Often, reduced use of acute healthcare services, such as fewer hospitalizations, fewer ICU admissions, and more home deaths, are considered quality indicators of hospice and palliative care [26]. Child- and family-centered quality indicators of palliative care should be adopted, regardless of the location of care and/or death, including systematic care planning, expressive therapies [2], encouraging normalcy, and independence for adolescents [27]. Furthermore, our results that ICU deaths were consistent regardless of palliative care involvement emphasize the importance of integrating palliative care into ICU settings.

### Palliative care involvement

Although it is difficult to directly compare the referral rate owing to the varying roles of the SPC team in patient care, the SPC referral rate of 60% was relatively high within the wide range reported in the previous literature [28, 29]. Rather, the overall involvement of SPC was probably underestimated because patients who were discharged under hospice care or transferred to another children’s hospital before death were not included in our analysis.

This study demonstrated that most children and young adult patients with malignancy received SPC, whereas those with premature and neonatal disease or cardiovascular disease did not. SPC involvement was less common in patients who died in the NICU and in younger patients, which was similar to a previous study [28]. The limited utilization of SPC in the NICU could be attributed to various barriers, including the complex and uncertain nature of the diseases, the lack of education and awareness among healthcare professionals, and the lack of institutional policies [30]. Our findings reveal missed opportunities to integrate palliative care into the NICU, as palliative care for neonates may benefit babies, parents, and healthcare professionals in pain and symptom management, decision-making and collaboration with parents, and psychological support [31].

### Strength and weaknesses

This study contributes to our understanding of the demographic and clinical characteristics of children and young adults who died in the hospital across different age groups as well as diverse disease groups. Furthermore, by comparing in-hospital deaths before and after the implementation of SPC, our findings address the impact of SPC on the acculturation of the general palliative approach within the children’s hospital.

This study has some limitations. First, the retrospective nature depends on complete documentation and accurate data retrieval, which makes it vulnerable, as it may depend on the provider and data collection process. However, this study utilized an automatic data-retrieval process to minimize these weaknesses. Second, patients who were cared for at the children’s hospital and died outside the hospital were excluded from the analyses due to limited access to the dataset. In the post-period, 59 patients died outside the hospital while maintaining SPC involvement. Therefore, our study has the potential to underestimate the impact of the SPC intervention on end-of-life healthcare utilization. Further studies are needed to investigate the effect of the SPC on end-of-life care, including deaths outside the hospital, to investigate the complete nature of SPC’s impacts on end-of-life care. Finally, this study investigated a single tertiary children’s hospital, which limits the generalizability of our findings to other institutions in different healthcare contexts. Given the substantial variation in the operations and structures of SPC programs across hospitals [32], the results from the robust SPC program at SNUCH may be challenging to generalize to settings with more limited PPC resources. Further multi-institutional prospective studies are required to validate the results of our study.

## Conclusions

This study demonstrated that patients with SPC tended to discuss care plans more and earlier and received less intensive care and more opioids at the end-of-life. This indicates more preparation for end-of-life care and proactive symptom control that reflects the patient’s and their family’s values and preferences. SPC seems to benefit not only the recipients of palliative care but also the institutional culture, fostering more and earlier ACP for patients with or without palliative care support. More research is warranted to investigate barriers and facilitators to integrating specialized palliative care for infants in the initial stage of palliative care implementation.

### Electronic supplementary material

Below is the link to the electronic supplementary material.

Supplementary Material 1

## Data Availability

Data from this study are available from the corresponding author upon reasonable request.

## Abbreviations

SPC: specialized palliative care
ACP: advance care planning
ED: emergency department
ICU: intensive care unit
SNUCH: Seoul National University Children’s Hospital
SUPREME: Seoul National University Hospital Patient Research Environment
OPD: outpatient department
NICU: neonatal intensive care unit
PICU: pediatric intensive care unit
SD: standard deviation
IQR: Interquartile range
LST: life-sustaining treatment
HD: hemodialysis
PD: peritoneal dialysis
IV: intravenous
PO: per oral
CPR: cardiopulmonary resuscitation
SE: standard error
CI: confidence interval
NHI: National Health Insurance
CCC: complex chronic condition
aOR: adjusted odds ratio
