# Supplementary material for: The impact of specialized pediatric palliative care on advance care planning and healthcare utilization in children and young adults: a retrospective analysis of medical records of in-hospital deaths
Source: BMC Palliat Care. 2024 May 22;23:127. doi: 10.1186/s12904-024-01448-w (PMC11110344; doi:10.1186/s12904-024-01448-w)
Supplement: Supplementary file 1 — Supplementary Material 1 [file 12904_2024_1448_MOESM1_ESM.docx]

Supplementary Table 1. Demographic characteristics of children and young adults who died at the children’s hospital compared to those who died elsewhere, among patients enrolled in specialized palliative care (n = 182)

| Characteristics | Total  (n = 182) | | In-hospital death  (n = 123) | | | Outside-hospital death  (n = 59) | | | *p*-value |
| --- | --- | --- | --- | --- | --- | --- | --- | --- | --- |
|  |  | | N (%), mean±SD, or median (IQR) | | | | | |  |
| Sex |  |  |  |  |  |  |  |  | .290 |
| Female | 59 | (22.3) | 43 | (35.0) |  | 16 | (27.1) |  |  |
| Male | 123 | (46.6) | 80 | (65.0) |  | 43 | (72.9) |  |  |
| Age at death, year | 9.2 | ± 8.6 | 6.8 | ± 7.1 | 4 (0, 13) | 11.6 | ± 10.0 | 9 (3, 20) | .001 |
| Insurance |  |  |  |  |  |  |  |  | .178 |
| NHI | 170 | (64.4) | 117 | (95.1) |  | 53 | (89.8) |  |  |
| Other | 12 | (4.5) | 6 | (4.9) |  | 6 | (10.2) |  |  |
| Resident area |  |  |  |  |  |  |  |  | .674 |
| Metropolitan near the hospital | 115 | (43.6) | 79 | (64.2) |  | 36 | (61.0) |  |  |
| Other | 67 | (25.4) | 44 | (35.8) |  | 23 | (39.0) |  |  |
| CCC diagnoses^a^ |  |  |  |  |  |  |  |  |  |
| Cardiovascular | 53 | (29.1) | 44 | (35.8) |  | 9 | (15.3) |  | .007 |
| Malignancy | 82 | (45.1) | 53 | (43.1) |  | 29 | (49.2) |  | .542 |
| Congenital or genetic | 29 | (15.9) | 25 | (20.3) |  | 4 | (6.8) |  | .034 |
| Premature or neonatal | 16 | (8.8) | 13 | (10.6) |  | 3 | (5.1) |  | .355 |
| Neurological and neuromuscular | 52 | (28.6) | 32 | (26.0) |  | 20 | (33.9) |  | .354 |
| Hematological or immunological | 56 | (30.8) | 47 | (38.2) |  | 9 | (15.3) |  | .003 |
| Metabolic | 19 | (10.4) | 12 | (9.8) |  | 7 | (11.9) |  | .860 |
| Gastrointestinal | 18 | (9.9) | 16 | (13.0) |  | 2 | (3.4) |  | .077 |
| Renal | 20 | (11.0) | 16 | (13.0) |  | 4 | (6.8) |  | .315 |
| Respiratory | 15 | (8.2) | 13 | (10.6) |  | 2 | (3.4) |  | .149 |
| Number of CCCs |  |  |  |  |  |  |  |  | <.001 |
| 0 | 8 | (4.4) | 3 | (2.4) |  | 5 | (8.5) |  |  |
| 1 | 64 | (35.2) | 34 | (27.6) |  | 30 | (50.8) |  |  |
| 2 | 62 | (34.1) | 47 | (38.2) |  | 15 | (25.4) |  |  |
| >3 | 48 | (26.4) | 39 | (31.7) |  | 9 | (15.3) |  |  |

SD, standard deviation; IQR, interquartile range; NHI, National Health Insurance; CCC, complex chronic condition.
^a^ Multiple responses were allowed.

Supplementary Table 2. Multivariable logistic analysis of confounding factors associated with the end-of-life care with post-period as an independent variable (n=479)

|  | Advance Care Planning  (legal documentation) | | | Opioid use | | | Death in general ward | | | Mechanical ventilation | | | Cardiopulmonary resuscitation | | | ICU admission | | |
| --- | --- | --- | --- | --- | --- | --- | --- | --- | --- | --- | --- | --- | --- | --- | --- | --- | --- | --- |
| Characteristics | aOR | (95% CI) | | aOR | (95% CI) | | aOR | (95% CI) | | aOR | (95% CI) | | aOR | (95% CI) | | aOR | (95% CI) | |
| Post-period^a^ | 1.62 | (1.08 | to 2.46)^*^ | 1.89 | (1.19 | to 3.06)^**^ | 1.54 | (0.85 | to 2.81) | 0.37 | (0.19 | to 0.68)^**^ | 0.45 | (0.30 | to 0.68)^***^ | 0.35 | (0.19 | to 0.64)^**^ |
| Male^a^ | 1.14 | (0.77 | to 1.69) | 1.20 | (0.78 | to 1.85) | 1.38 | (0.81 | to 2.40) | 0.63 | (0.35 | to 1.11) | 0.70 | (0.47 | to 1.03) | 0.88 | (0.51 | to 1.50) |
| Infant^a^ | 0.53 | (0.32 | to 0.88)^*^ | 2.73 | (1.56 | to 4.84)^***^ | 0.23 | (0.11 | to 0.48)^***^ | 8.86 | (3.85 | to 22.86)^***^ | 1.38 | (0.84 | to 2.27) | 5.74 | (2.80 | to 12.54)^***^ |
| Residence |  |  |  |  |  |  |  |  |  |  |  |  |  |  |  |  |  |  |
| Metropolitan near the hospital | 1.00 | (0.65 | to 1.54) | 0.82 | (0.50 | to 1.33) | 1.33 | (0.74 | to 2.41) | 0.73 | (0.39 | to 1.34) | 1.05 | (0.68 | to 1.62) | 0.72 | (0.39 | to 1.30) |
| Others | reference | | | — | | | — | | | — | | | — | | | — | | |
| Insurance type |  |  |  |  | | |  | | |  | | |  | | |  | | |
| NHI | 1.72 | (0.69 | to 4.57) | 2.41 | (0.75 | to 10.84) | 0.36 | (0.08 | to 1.26) | 3.02 | (0.76 | to 20.37) | 0.87 | (0.34 | to 2.17) | 3.47 | (0.89 | to 23.08) |
| Others | reference | | | — | | | — | | | — | | | — | | | — | | |
| CCC diagnosis |  |  |  |  |  |  |  |  |  |  |  |  |  |  |  |  |  |  |
| Cardiovascular^a^ | 1.06 | (0.49 | to 2.30) | 1.45 | (0.58 | to 3.77) | 1.10 | (0.43 | to 2.84) | 0.61 | (0.23 | to 1.62) | 0.82 | (0.35 | to 1.84) | 0.79 | (0.30 | to 2.07) |
| Malignancy^a^ | 1.45 | (0.60 | to 3.57) | 1.40 | (0.49 | to 4.19) | 6.87 | (2.39 | to 20.71)^***^ | 0.22 | (0.07 | to 0.64)^**^ | 0.35 | (0.13 | to 0.90)^*^ | 0.20 | (0.07 | to 0.60)^**^ |
| Congenital or genetic^a^ | 0.87 | (0.36 | to 2.09) | 1.10 | (0.39 | to 3.26) | 1.11 | (0.41 | to 3.06) | 0.67 | (0.23 | to 1.90) | 0.66 | (0.24 | to 1.65) | 0.44 | (0.15 | to 1.26) |
| Premature or neonatal^a^ | 0.83 | (0.36 | to 1.94) | 0.56 | (0.21 | to 1.58) | 0.25 | (0.04 | to 1.06) | 2.30 | (0.51 | to 16.35) | 0.89 | (0.35 | to 2.17) | 7.74 | (1.34 | to 147.37) |
| Neurological and neuromuscular^a^ | 0.97 | (0.40 | to 2.41) | 0.54 | (0.19 | to 1.58) | 3.07 | (1.02 | to 9.38)^*^ | 0.29 | (0.09 | to 0.87)^*^ | 0.57 | (0.21 | to 1.46) | 0.29 | (0.09 | to 0.89)^*^ |
| Hematological or immunological^a^ | 1.17 | (0.50 | to 2.83) | 3.47 | (1.19 | to 11.59)^*^ | 0.96 | (0.34 | to 2.67) | 1.22 | (0.43 | to 3.55) | 0.60 | (0.22 | to 1.48) | 1.07 | (0.37 | to 3.06) |
| Metabolic^a^ | 1.13 | (0.45 | to 2.88) | 0.73 | (0.25 | to 2.18) | 1.63 | (0.48 | to 5.44) | 0.57 | (0.16 | to 2.11) | 0.89 | (0.33 | to 2.37) | 0.57 | (0.16 | to 2.08) |
| Gastrointestinal^a^ | 0.68 | (0.26 | to 1.74) | 0.59 | (0.20 | to 1.77) | 0.85 | (0.26 | to 2.79) | 0.83 | (0.25 | to 2.80) | 0.66 | (0.23 | to 1.76) | 0.76 | (0.22 | to 2.55) |
| Renal^a^ | 1.01 | (0.42 | to 2.49) | 1.14 | (0.41 | to 3.49) | 1.09 | (0.36 | to 3.20) | 0.91 | (0.30 | to 2.88) | 0.68 | (0.26 | to 1.72) | 0.58 | (0.19 | to 1.74) |
| Respiratory^a^ | 0.66 | (0.26 | to 1.64) | 0.83 | (0.30 | to 2.38) | 2.30 | (0.69 | to 7.80) | 0.62 | (0.17 | to 2.31) | 0.83 | (0.31 | to 2.15) | 0.40 | (0.11 | to 1.39) |
| Number of CCCs |  |  |  |  |  |  |  |  |  |  |  |  |  |  |  |  |  |  |
| 0 | reference | | | — | | | — | | | — | | | — | | | — | | |
| 1 | 2.40 | (0.85 | to 6.95) | 3.36 | (1.09 | to 10.15)^*^ | 1.54 | (0.34 | to 8.65) | 0.99 | (0.20 | to 4.37) | 1.06 | (0.38 | to 3.06) | 2.64 | (0.70 | to 9.81) |
| 2 | 2.65 | (0.54 | to 12.81) | 3.59 | (0.55 | to 21.48) | 1.60 | (0.20 | to 14.05) | 1.21 | (0.14 | to 9.60) | 2.13 | (0.42 | to 12.34) | 3.66 | (0.53 | to 26.59) |
| ≥3 | 4.03 | (0.34 | to 46.64) | 4.52 | (0.23 | to 78.02) | 0.96 | (0.04 | to 20.64) | 3.15 | (0.14 | to 72.09) | 2.30 | (0.18 | to 36.13) | 11.23 | (0.57 | to 251.33) |
| Adjusted R^2^ | 0.107 | | | 0.141 | | | 0.291 | | | 0.255 | | | 0.111 | | | 0.262 | | |

aOR, adjusted odds ratio; CI, confidence interval; ICU, intensive care unit; NHI, national health insurance; CCC, complex chronic condition.
^a^ reference = no
*, p< .05; **, p< .01; ***, p< .001

Supplementary Table 3. Multivariable logistic analysis of the confounding factors associated with the end-of-life care with SPC group as an independent variable (n=205)

|  | Advance Care Planning  (legal documentation) | | | Opioid use | | | Death in general ward | | | Mechanical ventilation | | | Cardiopulmonary resuscitation | | | ICU admission | | |
| --- | --- | --- | --- | --- | --- | --- | --- | --- | --- | --- | --- | --- | --- | --- | --- | --- | --- | --- |
| Characteristics | aOR | (95% CI) | | aOR | (95% CI) | | aOR | (95% CI) | | aOR | (95% CI) | | aOR | (95% CI) | | aOR | (95% CI) | |
| SPC group^a^ | 5.47 | (2.53 | to 12.31)^***^ | 19.18 | (5.64 | to 82.61)^***^ | 6.22 | (1.61 | to 32.56)^*^ | 0.57 | (0.16 | to 1.89) | 0.18 | (0.08 | to 0.41)^***^ | 0.51 | (0.15 | to 1.55) |
| Male^a^ | 0.85 | (0.43 | to 1.68) | 1.82 | (0.75 | to 4.52) | 1.30 | (0.52 | to 3.32) | 0.47 | (0.18 | to 1.15) | 1.35 | (0.67 | to 2.76) | 0.91 | (0.38 | to 2.12) |
| Infant^a^ | 0.90 | (0.39 | to 2.11) | 2.87 | (0.92 | to 9.80) | 0.22 | (0.06 | to 0.72)^*^ | 9.26 | (2.74 | to 39.29)^**^ | 0.66 | (0.26 | to 1.61) | 4.88 | (1.77 | to 14.79)^**^ |
| CCC diagnosis |  |  |  |  |  |  |  |  |  |  |  |  |  |  |  |  |  |  |
| Cardiovascular^a^ | 0.79 | (0.26 | to 2.38) | 0.84 | (0.18 | to 4.17) | 0.50 | (0.12 | to 2.01) | 0.62 | (0.14 | to 2.49) | 0.65 | (0.18 | to 2.17) | 0.80 | (0.18 | to 3.26) |
| Malignancy^a^ | 1.02 | (0.27 | to 4.05) | 0.46 | (0.05 | to 4.68) | 6.00 | (1.31 | to 30.23)^*^ | 0.08 | (0.01 | to 0.41)^**^ | 0.49 | (0.10 | to 2.21) | 0.11 | (0.02 | to 0.54)^**^ |
| Congenital or genetic^a^ | 0.46 | (0.12 | to 1.86) | 0.41 | (0.05 | to 3.83) | 0.88 | (0.17 | to 4.53) | 0.52 | (0.09 | to 2.68) | 2.07 | (0.39 | to 10.33) | 0.37 | (0.06 | to 2.01) |
| Premature or neonatal^a^ | 0.77 | (0.25 | to 2.49) | 0.46 | (0.10 | to 2.35) | 0.15 | (0.01 | to 1.15) | 4.04 | (0.52 | to 88.95) | 1.09 | (0.28 | to 3.78) | 3.2e+07 | (0.00 | to 1.7e+210) |
| Neurological and neuromuscular^a^ | 0.65 | (0.19 | to 2.38) | 0.07 | (0.01 | to 0.52)^**^ | 2.29 | (0.47 | to 11.97) | 0.17 | (0.03 | to 0.84)^*^ | 0.33 | (0.07 | to 1.41) | 0.21 | (0.03 | to 1.05) |
| Hematological or immunological^a^ | 1.23 | (0.34 | to 4.68) | 8.53 | (1.02 | to 112.57) | 0.58 | (0.11 | to 2.95) | 1.29 | (0.22 | to 7.15) | 0.28 | (0.06 | to 1.25) | 0.84 | (0.14 | to 4.53) |
| Metabolic^a^ | 1.86 | (0.41 | to 9.19) | 0.47 | (0.05 | to 4.26) | 2.44 | (0.37 | to 16.62) | 0.25 | (0.03 | to 1.84) | 1.27 | (0.23 | to 6.61) | 0.23 | (0.03 | to 1.59) |
| Gastrointestinal^a^ | 0.92 | (0.22 | to 3.98) | 0.64 | (0.07 | to 5.36) | 0.48 | (0.08 | to 2.70) | 0.34 | (0.05 | to 1.83) | 0.69 | (0.13 | to 3.31) | 0.63 | (0.09 | to 3.71) |
| Renal^a^ | 1.10 | (0.30 | to 4.36) | 0.26 | (0.04 | to 1.79) | 0.37 | (0.06 | to 1.97) | 1.42 | (0.25 | to 8.22) | 0.25 | (0.04 | to 1.19) | 1.11 | (0.20 | to 6.35) |
| Respiratory^a^ | 0.56 | (0.14 | to 2.17) | 2.11 | (0.32 | to 17.56) | 2.19 | (0.39 | to 12.81) | 0.21 | (0.03 | to 1.29) | 1.07 | (0.23 | to 4.59) | 0.43 | (0.06 | to 2.64) |
| CCC number |  |  |  |  |  |  |  |  |  |  |  |  |  |  |  |  |  |  |
| 0 | reference | | | — | | | — | | | — | | | — | | | — | | |
| 1 | 2.05 | (0.40 | to 11.61) | 8.41 | (1.15 | to 63.03)^*^ | 1.59 | (0.13 | to 41.41) | 2.62 | (0.23 | to 25.85) | 1.26 | (0.24 | to 7.00) | 11.3 | (1.54 | to 95.30)^*^ |
| 2 | 2.36 | (0.22 | to 24.63) | 11.83 | (0.48 | to 272.41) | 1.82 | (0.07 | to 73.76) | 2.93 | (0.13 | to 75.81) | 4.19 | (0.35 | to 63.78) | 16.45 | (0.88 | to 423.20) |
| ≥3 | 4.06 | (0.10 | to 164.51) | 21.53 | (0.10 | to 4742.20) | 2.92 | (0.02 | to 391.37) | 10.09 | (0.10 | to 1565.92) | 3.31 | (0.06 | to 267.68) | 53.73 | (0.54 | to 9109.13) |
| Adjusted R^2^ | 0.213 | | | 0.333 | | | 0.403 | | | 0.374 | | | 0.229 | | | 0.349 | | |

aOR, adjusted odds ratio; CI, confidence interval; ICU, intensive care unit; NHI, national health insurance; CCC, complex chronic condition.
^a^ reference = no
*, p< .05; **, p< .01; ***, p< .001

Table 4. Days from initial discussion of advance care planning to death, by demographic and clinical characteristics (n=169)

| Characteristics | n | (%) | mean | ± SD |
| --- | --- | --- | --- | --- |
| Total | 169 | (100.0) | 37.6 | ± 79.3 |
| Sex |  |  |  |  |
| Female | 65 | (38.5) | 39.5 | ± 89.9 |
| Male | 104 | (61.5) | 36.4 | ± 72.4 |
| Age at death 12 months or younger |  |  |  |  |
| No | 89 | (52.7) | 60.4 | ± 99.6 |
| Yes (infant) | 80 | (47.3) | 12.3 | ± 33.1 |
| Residence |  |  |  |  |
| Others | 59 | (34.9) | 34.3 | ± 74.6 |
| Metropolitan near the hospital | 110 | (65.1) | 39.4 | ± 82.1 |
| Insurance type |  |  |  |  |
| Others | 7 | (4.1) | 17.1 | ± 22.5 |
| NHI | 162 | (95.9) | 38.5 | ± 80.8 |
| SPC involvement |  |  |  |  |
| Non-SPC group | 53 | (31.4) | 4.8 | ± 14.3 |
| SPC-group | 116 | (68.6) | 52.6 | ± 91.5 |
| Malignancy |  |  |  |  |
| No | 116 | (95.9) | 31.4 | ± 81.1 |
| Yes | 53 | (4.1) | 51.2 | ± 74.2 |
| Neurological and Neuromuscular |  |  |  |  |
| No | 134 | (79.3) | 29.4 | ± 70.0 |
| Yes | 35 | (20.7) | 69.2 | ± 103.0 |
